# Angiotensin II type 2 receptor (AT_2_R) localization and antagonist-mediated inhibition of capsaicin responses and neurite outgrowth in human and rat sensory neurons

**DOI:** 10.1002/j.1532-2149.2012.00269.x

**Published:** 2012-12-17

**Authors:** U Anand, P Facer, Y Yiangou, M Sinisi, M Fox, T McCarthy, C Bountra, YE Korchev, P Anand

**Affiliations:** 1 Peripheral Neuropathy Unit, Department of Clinical Neuroscience, Imperial College LondonUK; 2 Nanoscience Research Laboratory, Division of Medicine, Imperial College LondonUK; 3 Peripheral Nerve Injury Unit, Royal National Orthopaedic HospitalStanmore, UK; 4 Spinifex Pharmaceuticals Pty LtdVictoria, Australia; 5 University of Oxford Structural Genomics ConsortiumUK

## Abstract

**Background:**

The angiotensin II (AngII) receptor subtype 2 (AT_2_R) is expressed in sensory neurons and may play a role in nociception and neuronal regeneration.

**Methods:**

We used immunostaining with characterized antibodies to study the localization of AT_2_R in cultured human and rat dorsal root ganglion (DRG) neurons and a range of human tissues. The effects of AngII and AT_2_R antagonist EMA401 on capsaicin responses in cultured human and rat (DRG) neurons were measured with calcium imaging, on neurite length and density with Gap43 immunostaining, and on cyclic adenosine monophosphate (cAMP) expression using immunofluorescence.

**Results:**

AT_2_R expression was localized in small-/medium-sized cultured neurons of human and rat DRG. Treatment with the AT_2_R antagonist EMA401 resulted in dose-related functional inhibition of capsaicin responses (IC_50_ = 10 nmol/L), which was reversed by 8-bromo-cAMP, and reduced neurite length and density; AngII treatment significantly enhanced capsaicin responses, cAMP levels and neurite outgrowth. The AT_1_R antagonist losartan had no effect on capsaicin responses. AT_2_R was localized in sensory neurons of human DRG, and nerve fibres in peripheral nerves, skin, urinary bladder and bowel. A majority sub-population (60%) of small-/medium-diameter neuronal cells were immunopositive in both control post-mortem and avulsion-injured human DRG; some very small neurons appeared to be intensely immunoreactive, with TRPV1 co-localization. While AT_2_R levels were reduced in human limb peripheral nerve segments proximal to injury, they were preserved in painful neuromas.

**Conclusions:**

AT_2_R antagonists could be particularly useful in the treatment of chronic pain and hypersensitivity associated with abnormal nerve sprouting.

## 1. Introduction

The octapeptide angiotensin II (AngII) is known to regulate blood pressure, fluid balance and other functions via two known membrane bound G protein-coupled receptors, angiotensin II type 1 receptor (AT_1_R) and angiotensin II type 2 receptor (AT_2_R) (De Gasparo et al., 2000; Paul et al., 2006). There is increasing evidence that AngII may play a significant role in the nervous system, including pain mechanisms. AngII and AT_2_R protein expression have been detected in rat dorsal root ganglion (rDRG), human dorsal root ganglion (hDRG) and trigeminal ganglia (Chakrabarty et al., 2008; Imboden et al., 2009; Patil et al., 2002), and AT_2_R mRNA in hDRG extracts, indicating the existence of an intrinsic angiotensinergic system. Furthermore, co-localization of AngII with substance P and calcitonin gene-related peptide containing DRG neurons (Patil et al., 2002) suggests a role for AngII in nociception. AT_2_R antagonists have shown efficacy in rodent neuropathic pain models (see Smith, 2011; Smith and Wyse, 2011), and the clinical efficacy and safety of AT_2_R antagonist EMA401 was reported recently in post-herpetic neuralgia (McCarthy et al., 2012).

Neurite-promoting effects of AngII have been described in the optic nerve of adult rats (Lucius et al., 1998), cerebellar explants (Cote et al., 1999) and NG108-15 cells (Plouffe et al., 2006; Wallinder et al., 2008; Guimond et al., 2010), and in functional recovery after sciatic nerve damage in rats (Reinecke et al., 2003). The neurite-promoting effect was observed in oestrogen-treated small/medium cultured rDRG neurons, which was eliminated by AT_2_R blockade, indicating a potential modulatory role in both pain signalling and neurite regeneration (Chakrabarty et al., 2008).

While AngII and its metabolite AngIII both act at the AT_1_R and the AT_2_R in the brain (Zini et al., 1996; Wright et al., 2003, Pelegrini-Da-Silva et al., 2005), and have important effects in the central nervous system (CNS) on pain mechanisms (see the Discussion section), we have focused on peripheral mechanisms since the AT_2_R antagonist EMA401 used in our study does not have significant CNS distribution after oral dosing.

**What's already known about this topic?**

- The angiotensin II type 2 receptor (AT_2_R) is expressed in sensory neurons, and in rat DRG AT2R mRNA co-localises with substance P, suggesting an involvement in nociception.

**What this study adds?**

- The AT2R is expressed in human peripheral somatic and visceral nerves, and is co-localised with TRPV1 in human DRG neurons. The AT_2_R antagonist EMA401 inhibits capsaicin responses and angiotensin II (AngII)-induced cyclic adenosine monophosphate (cAMP) increases in human and rat cultured DRG neurons. AngII causes calcium influx in DRG neurons and sensitizes capsaicin-mediated calcium influx.

We have examined the functional effects of the AT_2_R antagonist EMA401 in cultured human and rat DRG neurons on the responses to capsaicin. EMA401 is a member of the tetrahydroisoquinoline class of AT_2_R antagonists (Supporting Information Fig. S1). Capsaicin is the pungent ingredient of chilli peppers, which acts on the TRPV1 receptor in nociceptive neurons (Smith et al., 2002; Facer et al., 2007) to activate calcium influx, leading to the sensation of pain. TRPV1 is activated by a variety of noxious stimuli, including capsaicin, heat, protons and leukotrienes (Tominaga et al., 1998; Hwang et al., 2010). Furthermore, there is evidence that shows that TRPV1 expression is up-regulated in clinical conditions of chronic pain (Mathews et al., 2004; Apostolidis et al., 2005; Yilmaz et al., 2007; Akbar et al., 2008). We have used an *in vitro* model described previously (Anand et al., 2006, 2010) for determining the antinociceptive effects of EMA401 on capsaicin responses and its morphological effects on neurite outgrowth. We also studied the expression of AT_2_R in a range of normal and post-nerve injury human tissues using immunocytochemistry.

## 2. Methods

### 2.1. In vitro studies

Preparation of cultured neurons was as described previously; briefly, avulsed human cervical DRG (hDRG) were obtained as a necessary part of the surgical nerve repair procedure from three patients, with fully informed consent and approval of the Local Research Ethics Committee, Royal National Orthopaedic Hospital, Stanmore, UK. Tissue was enzyme-digested and mechanically dissociated to yield a neuronal suspension, which was plated on collagen and laminin-coated glass bottomed MatTek dishes (MatTek Corp., Ashland, MA, USA) in Ham's F12 medium containing 10% heat-inactivated fetal calf serum (HIFCS), penicillin and streptomycin (100 μg/mL each), and neurotrophic factors (NTFs) such as nerve growth factor (NGF) (100 ng/mL), GDNF and NT3 (50 ng/mL each). Cultures were incubated at 37 °C in a humid environment for 48 h before being treated with AngII, or EMA401 for determining the effects on neurite density or used for Ca^2+^ imaging studies. Similarly, bilateral DRG from all levels were harvested from 12 adult female Wistar rats (Charles River UK Ltd, Margate, Kent, UK) and neuronal cultures were prepared as described above, and incubated in BSF2 medium [containing 2% HIFCS, 0.1 mg/mL transferrin, 60 ng/mL progesterone, 0.16 μg/mL sodium selenite, 3 mg/mL bovine serum albumen (BSA), penicillin/streptomycin 100 μg/mL each, 16 μg/mL putrescine, 10 μg/mL insulin], and NTFs for 48 h before being studied.

#### 2.1.1. Calcium imaging

Functional effects of acute EMA401 treatment on capsaicin responses were determined as previously described in Fura2 AM (Molecular Probes Life Technologies, Paisley, UK) loaded neurons (Anand et al., 2006, 2008). Responses to paired capsaicin stimuli, with and without EMA401, AngII or 8-bromo-cAMP (Calbiochem, Merck Chemicals Ltd, Beeston, Nottingham, UK), were measured in Fura2 AM loaded neurons as a change in the baseline 340/380λ_ex_ nm ratio before, during and after addition. Experiments were conducted at 37 °C in a humidified environment on an inverted Nikon microscope (Diaphot 300; Nikon, UK Ltd, Kingston Upon Thames, Surrey, UK) and alternately excited at 340 and 380 nm wavelengths. Forskolin was used as a surrogate source of cyclic adenosine monophosphate (cAMP) to test for adenylyl cyclase involvement, and confirmed with 8-bromo cAMP (details in Supporting Information Method S1).

#### 2.1.2. cAMP assay

Duplicate dishes of neurons were treated with 10 nmol/L AngII, 10 nmol/L AngII plus 100 nmol/L EMA401, 50 μmol/L 8-bromo-cAMP (positive control), 100 nmol/L EMA401 or vehicle (0.2% v/v HBSS) controls, for 30 min, before 4% paraformaldehyde (PFA) fixation for 15 min and immunostaining.

#### 2.1.3. Neurite outgrowth assay

Neuronal cultures were treated with AngII, EMA401 or both, and compared with NTF-treated controls in duplicate for 48 h, followed by 4% PFA fixation and Gap43 immunostaining. Lyophylized AngII (Sigma, Poole, UK) was reconstituted in sterile distilled water, aliquoted and stored at −20 °C. EMA401 and Losartan (Sigma) were dissolved in HBSS containing 0.1% BSA (pH 7.4) and stored at 4 °C.

#### 2.1.4. Immunostaining and morphological assessment

Neurons were permeabilized with methanol (−20 °C, 3 min), washed with PBS and immunostained with primary antibodies to mGap43 (mouse monoclonal, 1: 200; Sigma), goat anti-AT_2_R (polyclonal, 1:200, sc-48452; Santa Cruz Biotechnology Inc., Santa Cruz, CA, USA), or rabbit anti-cAMP (AB306, 1:1000; Millipore, Billerica, MA, USA), and visualized with donkey anti-mouse IgG (Alexa 488, 1:200; Molecular Probes), donkey anti-goat IgG (Alexa 594, 1:150; Molecular Probes), goat anti-mouse IgG (Alexa 488, 1:200; Molecular Probes), or goat anti-rabbit IgG (Alexa 488, 1:200; Molecular Probes), 1 h each at room temperature. The glass bottom cover slips were mounted on glass slides in glycerol containing Hoechst dye 33342 and anti-fade agent DABCO [1,4-diazobicyclo-(2,2,2)-octane], and sealed with nail varnish. Tiff fluorescence images at a fixed exposure were acquired after setting the threshold levels, with an upright Olympus microscope (Olympus Medical, Essex, UK) using SmartCapture software (Digital Scientific, Cambridge, UK), after confirming the absence of immunostaining in negative controls where the primary antibody had been omitted. Diameters of AT_2_R positive and negative neurons were measured as the average of the widest and longest axes perpendicular to each other and plotted as a frequency distribution, and the average diameters for AT_2_R positive and negative neurons were calculated. Gap43 immunostained images were analysed for mean neurite density (mean fluorescence intensity) from individual neurons after background subtraction; averages of five measurements (arbitrary units) within a fixed area of 100 μm^2^ were obtained from the neurite-containing area surrounding each neuron, from 50 neurons in each preparation using ImageJ software (NIH, National Institutes of Health, Bethesda, MD, USA). Maximum neurite lengths were measured from Tiff images of individual neurons using ImageJ software. Similarly, Tiff images at a fixed exposure were acquired from neuronal cultures immunostained for cAMP, after confirming the absence of immunostaining in negative controls, in which the primary antibody had been omitted. After background subtraction, the mean fluorescence intensity in each neuron was measured from a demarcated circular region of interest from the brightest region in the cell body, and the values were averaged for each group, using ImageJ software. Neurite density, length and cAMP fluorescence intensity are expressed as percent of control ± standard error of the mean (SEM). Student's unpaired *t*-test was used to compare between groups; *p* < 0.05 was considered to be statistically significant.

### 2.2. Tissues

A range of tissues were used in this study for which fully informed consent was obtained with approval of the Local Ethics Committee. Specimens were snap-frozen in liquid nitrogen and stored at –70 °C until use or immersed in Zamboni's fixative (2% w/v formalin, 0.1 mol/L phosphate and 15% v/v saturated picric acid) for 2 h and stored in phosphate buffered saline (PBS) containing 15% sucrose and 0.01% azide.

### 2.3. DRG and peripheral nerves

Avulsed human DRG (*n* = 7, cervical; mean age years ± SEM = 31.5 ± 6.0; 1 women; injury delay range = 3 days–5 months) were obtained from patients having surgery for brachial plexus repair. Control, post-mortem DRG (*n* = 4; mean age years ± SEM = 69.5 ± 5.8; 2 women; mean post-mortem delay hours ± SEM = 10.3 ± 2.6) were obtained from the Netherlands Brain Bank. Specimens of proximal injured limb nerves (*n* = 9; mean age years ± SEM = 29.0 ± 4.8; 2 women; injury to surgery delay range = 1.5 days–12 months), painful neuromas (*n* = 25; mean age years ± SEM = 23.3 ± 2.8; 4 women; injury-to-surgery delay range = 1.5–13 months, pre-operative pain intensity scores >3/10 on 11-point pain intensity numerical rating scale) were obtained from patients having surgery for painful neuroma relocation or peripheral nerve repair. Uninjured nerves (*n* = 6; mean age years ± SEM = 41.6 ± 10.6; 2 women), used as grafts in nerve repairs during surgery, served as controls.

### 2.4. Skin

Skin samples (4-mm punch biopsies) were obtained under local anaesthesia from the leg (calf) of normal control subjects (*n* = 6; mean age 41 years, range 25–54 years; 4 women).

### 2.5. Urinary bladder

Tissue specimens (cystoscopic biopsies) were obtained from control subjects under investigation for asymptomatic microscopic haematuria (*n* = 12; mean age = 51 years; range = 31–79 years; 9 women).

### 2.6. Intestine

Samples of full thickness, surgically resected large bowel were obtained from patients undergoing surgery for carcinoma or disease unrelated to inflammation, where uninvolved normal regions were used as controls (*n* = 8; age mean age = 63 years; range = 17–77 years; 3 women).

### 2.7. Immunohistology

Tissues were supported in optimum cutting tissue (OCT) medium (RA Lamb Ltd, Eastbourne, UK). Tissue sections (15 μm thick) were collected onto poly-L-lysine (Sigma)-coated glass slides and post-fixed in 4% w/v PFA in 0.15 mol/L PBS for 30 min (for frozen section only). Endogenous peroxidase was blocked by incubation in methanol containing 0.3% w/v hydrogen peroxide for 30 min. After rehydration with PBS buffer, sections were incubated overnight with primary antibodies to rabbit anti-AT_1_ (sc-1173; Santa Cruz Biotechnology Inc.), goat anti-AT_2_R (sc-7420, sc-48451 and sc-48452; Santa Cruz Biotechnology Inc.), rabbit anti-AT_2_R (sc-9040; Santa Cruz Biotechnology Inc.; and Ab 19134, Abcam, Cambridge, UK) and monoclonal antibodies to neurofilament and peripherin cocktail (M0762 and NCL-periph; Dako UK Ltd, Cambridge, UK), using a range of dilutions. For immunostaining specificity, antibodies to AT_2_R (sc-48452) were incubated with AT_2_R peptide antigen, in the range of 5 × 10^−2^ to 5 × 10^−8^ mg/mL diluted (1/100) antibodies, prior to immunostaining in DRG.

Sites of primary antibody attachment were revealed using nickel-enhanced, avidin-biotin peroxidase (ABC; Vector Laboratories, Peterborough, UK) as described (Shu et al., 1988). Sections were counter-stained for nuclei in 0.1% w/v aqueous neutral red, dehydrated and mounted in xylene-based mount (DPX; BDH/Merck, Poole, UK) prior to photomicrography.

AT_2_R-immunoreactive, nucleated neurons in sensory ganglia (DRG) were counted throughout the sections and their diameter was assessed using a calibrated microscope eyepiece graticule. Nerve samples were assessed quantitatively using computerized image analysis from images captured using an Olympus DP70 camera mounted to an Olympus BX50 microscope, and analysed using analySIS (version 5.0 Soft Imaging System GmbH, Munster, Germany) software. Positive immunostaining was highlighted by setting the grey-level detection limits to threshold and the area of highlighted immunoreactivity obtained as % area of the field scanned and five random fields per tissue section were scanned at the same magnification (×40). Results were expressed as % area, and to correct for variation in overall nerve density, they were also expressed as the ratio AT_2_R : neurofilaments. The Mann–Whitney test was used for statistical analysis (*p*-values <0.05 were considered statistically significant).

For double immunofluorescence of AT_2_R and TRPV1 in control hDRG sections, 15-μm-thick frozen sections were fixed in 4% PFA for 20 min, and double-immunostained with the antibodies rabbit anti-TRPV1 (1:1000, GlaxoSmithKline, Harlow, Essex, UK) and goat anti-AT_2_R (1:200, sc-48452; Santa Cruz Biotechnology), visualized with donkey anti-rabbit (Alexa 488, 1:200; Molecular Probes) and donkey anti-goat (Alexa 594,1:200; Molecular Probes). Sections were mounted in glycerol containing DABCO (anti-fade agent) and TIFF fluorescence images were acquired with Olympus BX43 using Cellsense software (Olympus). The proportion of AT_2_R positive neurons and co-localization with TRPV1 was estimated.

### 2.8. Western blotting

Protein extracts were prepared by homogenizing tissue in standard RIPA buffer at 4 °C. Homogenates from human bladder and peripheral nerve were available for use with the AT_2_R antibody (details in Supporting Information Method S2).

## 3. Results

### 3.1. In vitro

**Figure 1:**
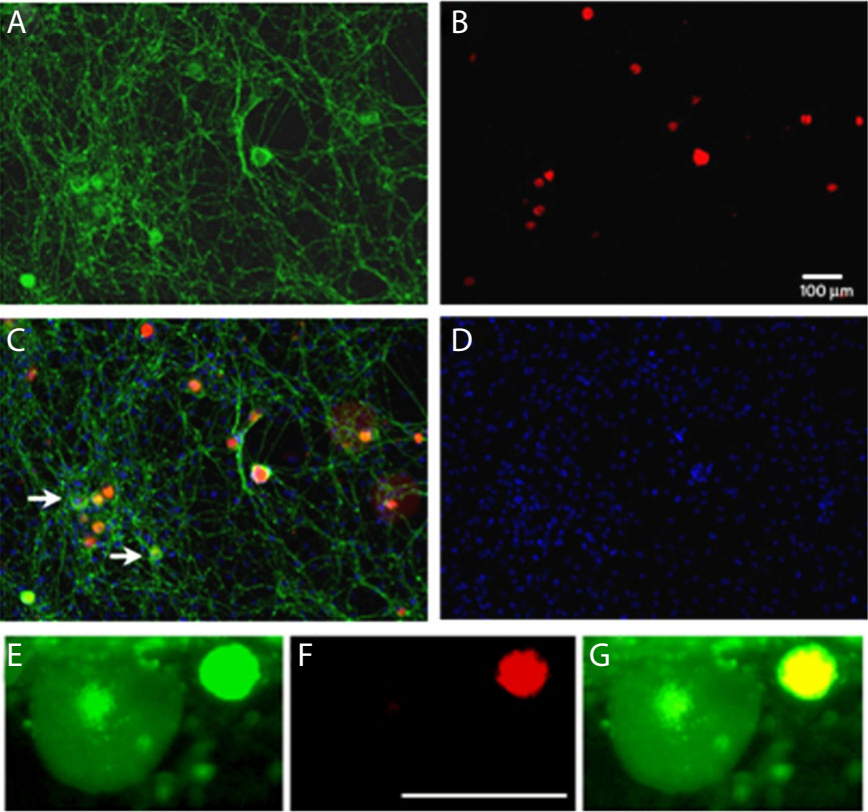
Low power image of cultured adult rat dorsal root ganglion (DRG) neurons showing Gap43 neurons and neurites (A), angiotensin II type 2 receptor (AT_2_R) positive neuronal cell bodies (B), and merged image with intense co-localization in some neurons (C, arrows indicate AT_2_R negative neurons), and nuclei (D, Hoechst dye positive); bar = 100 μm. High power image of cultured adult hDRG neurons positive for Gap43 (E), smaller neuron positive for AT_2_R (F), and merged image (G); bar in (F) = 50 μm.

rDRG neurons were observed to be uniformly labelled with Gap43 (Fig. 1A), smaller-sized neurons were double-labelled for AT_2_R and Gap43 (Fig. 1B and C). Cultured hDRG neurons were also double-labelled for Gap43 and AT_2_R (Fig. 1E–G); analysis of the cell body diameters showed that the AT_2_R positive neurons in hDRG were also of small diameter (average 36 ± 2.6 μm), while AT_2_R negative neurons had a larger average diameter of 55.9 ± 4 μm (measurements taken from 42 neurons).

### 3.2. Effect of AngII and EMA401 (AT_2_R antagonist) on capsaicin responses

**Figure 2:**
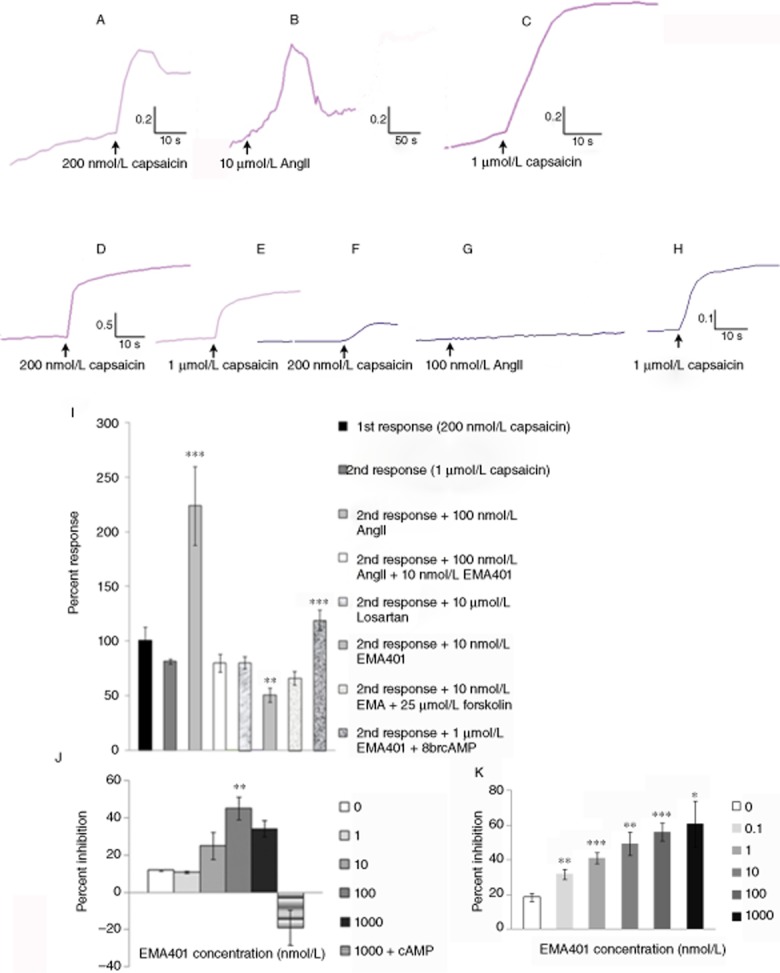
Sample traces showing calcium influx in human dorsal root ganglion (hDRG) neurons in response to 200 nmol/L capsaicin (first stimulus, A), followed by washout and response to 10 μmol/L AngII (B), followed by washout and increased response to second capsaicin stimulus (1 μmol/L) (C). Different hDRG neuron showing calcium influx in response to the first capsaicin stimulus (D), followed by washout, and reduced second response to capsaicin after 10 nmol/L EMA401 application (E). Rat dorsal root ganglion (rDRG) neuron showing response to first capsaicin stimulus (F), followed by washout and no response to 100 nmol/L AngII application (G), followed by enhanced second response to capsaicin (H). Graph (I) showing amplitude of capsaicin responses in the absence of drugs (bars 1 and 2), significant enhancement with AngII treatment (bar 3, ***p < 0.001, in comparison with bar 2), and abolition of this AngII-mediated enhancement with EMA401 application (bar 4). Losartan (AT1 antagonist) had no effect on capsaicin responses (bar 5). EMA401-mediated capsaicin response inhibition (bar 6, **p < 0.005, in comparison with bar 2) was reversed in the presence of forskolin (bar 7, in comparison with bar 6) and 8-bromo-cAMP (bar 8, in comparison with bar 6, ***p < 0.001). (J) shows dose-related inhibition of capsaicin responses in hDRG neurons by EMA401, and reversal with 8-bromo-cAMP (J). (K) shows dose-related capsaicin inhibition by EMA401 in rDRG neurons.

Representative traces are shown in Fig. 2A–H. Recordings of the 340/380 ratio showed a stable baseline, with a rapid increase in capsaicin stimulation in hDRG neuron (Fig. 2A). AngII application (100 nmol/L and 10 μmol/L) stimulated calcium influx in capsaicin responsive neurons of hDRG [response to 10 μmol/L AngII after first capsaicin application (200 nmol/mL), as shown in Fig. 2B] and significant enhancement of subsequent capsaicin response (Fig. 2C). Pre-incubation with EMA401 reduced the subsequent capsaicin response shown in a different hDRG neuron (response to capsaicin first stimulus, Fig. 2D; inhibitory effect of EMA401, Fig. 2E). In a rDRG neuron, sample traces demonstrate the response to the first capsaicin stimulus (Fig. 2F), lack of response on addition of 100 nmol/L AngII (Fig. 2G) and enhanced response to second capsaicin stimulus (Fig. 2H).

In Fig. 2I, responses in rDRG neurons to the first 200 nmol/L capsaicin stimulus are shown in bar 1 and to second 1 μmol/L capsaicin stimulus in bar 2 (note the expected tachyphylaxis). Furthermore, 100 nmol/L AngII treatment did not cause calcium influx by itself when added following the first capsaicin stimulus in rDRG neurons, but significantly enhanced subsequent capsaicin responses (bar 3, *n* = 5 experiments, 21 neurons, ***p < 0.001). This enhanced response following addition of AngII was inhibited in the presence of EMA401 (bar 4). Concentrations higher than 100 nmol/L AngII are required for direct calcium influx responses in capsaicin-sensitive rDRG neurons (studies in progress). The AT_1_ antagonist losartan (10 μmol/L) did not affect capsaicin responses (bar 5, *n* = 4 experiments, 11 neurons), but the second capsaicin response was significantly reduced by 10 nmol/L EMA401 (bar 6, *n* = 6 experiments, 24 neurons, **p < 0.005). Capsaicin inhibition by 10 nmol/L EMA401 was reversed in the presence of 25 μmol/L forskolin (bar 7, *n* = 3 experiments, 12 neurons, *p* < 0.05), and actually enhanced by excess of 8-bromo-cAMP (bar 8, *n* = 3 experiments, 20 neurons, ***p < 0.001).

In Fig. 2J, dose-related inhibition of capsaicin responses is shown in hDRG neurons (0 EMA401, *n* = 4 neurons; 1 nmol/L EMA401, *n* = 3 neurons; 10 nmol/L EMA401, *n* = 4 neurons; 100 nmol/L EMA401, *n* = 5 neurons; 1000 nmol/L EMA401, *n* = 3 neurons; IC_50_ = 10 nmol/L). Inhibition of capsaicin responses was reversed in the presence of 8-bromo-cAMP (bar 6, Fig. 2J). While application of 10 μmol/L AngII stimulated calcium influx after the first capsaicin stimulus and return to baseline, and 100 nmol/L AngII produced a small response, further dose–response studies of AngII in hDRG neurons, including effect without any prior capsaicin stimulation, are in progress.

In Fig. 2K, dose-related inhibition of capsaicin responses is shown following treatment with EMA401 in rDRG neurons (0 EMA401, *n* = 5 experiments, 21 neurons; 0.1 nmol/L EMA401, *n* = 5 experiments, 19 neurons, **p < 0.005; 1 nmol/L EMA401, *n* = 4 experiments, 16 neurons, ***p < 0.001; 10 nmol/L EMA401, *n* = 6 experiments, 24 neurons, **p < 0.005; 100 nmol/L EMA401, *n* = 6 experiments, 21 neurons, ***p < 0.001; 1000 nmol/L EMA401, *n* = 4 experiments, 10 neurons, *p < 0.05; IC_50_ = 10 nmol/L). In preliminary studies, inhibition of capsaicin responses by 100 nmol/L EMA401 (*n* = 6 experiments, 21 neurons, 56 ± 5%) was comparable to maximum inhibition by 1 μmol/L gabapentin [49.1 ± 3.2%, *n* = 3, 5 neurons, not significant (n.s.)] and 10 μmol/L morphine (67.22 ± 4.3% inhibition, *n* = 3, 3 neurons, n.s.).

### 3.3. Effect of AngII and EMA401 on neurite density

**Figure 3:**
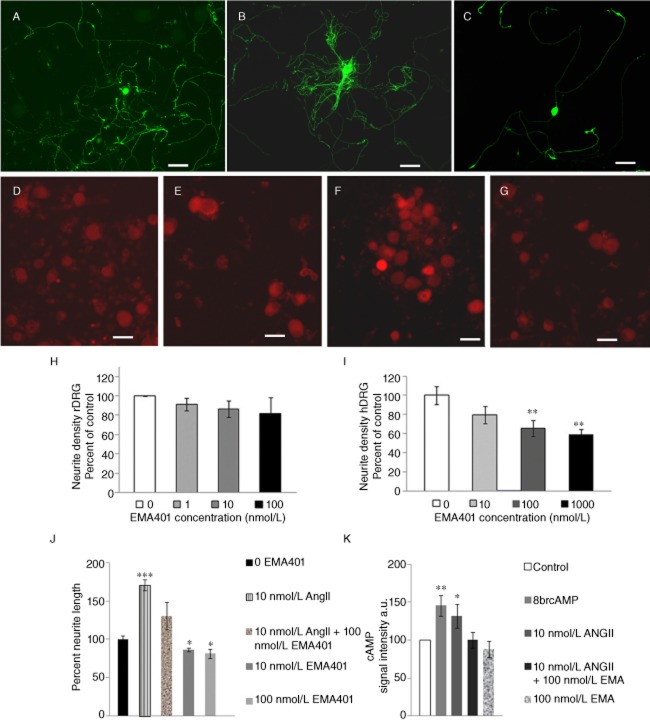
Morphology of Gap43-immunostained cultured rat dorsal root ganglion (rDRG) neurons without AngII treatment (A), with AngII treatment (B), and after treatment with 100 nmol/L EMA401 (C). Examples of cAMP positive, immunofluorescent neurons without AngII (D), with AngII treatment (E), with 8-bromo-cAMP (F), and with 100 nmol/L EMA401 (G). Scale bar in A–C = 50 μm; D–G = 30 μm. Graph showing neurite density in EMA401-treated rDRG neurons (H), significantly reduced neurite density in hDRG neurons treated with EMA401 (I; **p = 0.005. Graph (J) showing neurite length is significantly increased in AngII-treated rDRG neurons (bar 2, ***p < 0.001), but this not significant after EMA401 treatment (bar 3, J). 10 and 100 nmol/L EMA401 by itself reduced average neurite length compared with control neurons (bars 4 and 5, J; *p < 0.05). Graph (K) of cAMP immunofluorescence signal intensity showing significant increase after 8-br-cAMP treatment (bar 2, **p < 0.01), and after 10 nmol/L AngII treatment in rDRG neurons (bar 3, *p < 0.05). Neurons treated with a combination of 10 nmol/L AngII and 100 nmol/L EMA401 did not have significantly increased cAMP levels (bar 4, K). Treatment with 100 nmol/L EMA401 alone showed a slight reduction, but this was not significant (bar 5, K).

Gap43 immunostaining showed cell bodies and neurites (Fig. 3A); AngII treatment resulted in dense neurite outgrowth (Fig. 3B), while EMA401 treatment had an opposite effect (Fig. 3C). Neurites appeared to be healthy with no indication of vesicle formation or degeneration and were positively and uniformly immunostained in all preparations (Fig. 3A–C). In rDRG neurons treated with EMA401 (Fig. 3H), there was a tendency for mean neurite density to be reduced compared with untreated controls, but this was not statistically significant.

In hDRG neurons, mean neurite density was observed to be reduced after EMA401 treatment (Fig. 3I: 10 nmol/L, *n* = 12 neurons, *p* = 0.65 n.s.; 100 nmol/L, *n* = 10 neurons, **p = 0.005; and 1 μmol/L, *n* = 12 neurons, *p* = 0.005**) compared with controls (*n* = 14 neurons).

### 3.4. Effect of AngII and EMA401 on neurite length

Control rDRG neurons had an average neurite length of 717 ± 25 μm. Neurons treated with 10 nmol/L AngII had exuberant longer, denser neurites with a significantly increased average neurite length (bar 2, Fig. 3J: *n* = 3 experiments, ***p < 0.001), compared with controls; 100 nmol/L EMA401 reduced this AngII-mediated increase, which was no longer significant (bar 3, in comparison with bar 1). Furthermore, 10 and 100 nmol/L EMA401 by itself reduced the average neurite length compared with neurite length of control neurons by a small but significant extent (bars 4 and 5, Fig. 3J, *p < 0.05).

### 3.5. Effect of AngII and EMA401 treatment on cAMP expression

Control rDRG neurons showed cAMP immunofluorescence (Fig. 3D), which appeared to be increased after 10 nmol/L AngII (Fig. 3E) and 8-bromo-cAMP (Fig. 3F); the AngII-mediated increase appeared diminished by 100 nmol/L EMA401 (Fig. 3G). The cAMP antibody used does detect 8-bromo-cAMP (see Anand et al., 2010). Fig. 3K shows quantitative results; treatment with 8-bromo-cAMP showed a significant increase (bar 2, **p < 0.01) compared with controls (bar 1), and there were significantly increased levels of cAMP immunofluorescence by 10 nmol/L AngII (bar 3, *p < 0.05). Neurons treated with a combination of 10 nmol/L AngII and 100 nmol/L EMA401 did not have significantly increased cAMP levels (i.e., EMA401 prevented the increase; bar 4, Fig. 3K), and treatment with 100 nmol/L EMA401 alone showed a slight reduction, but this was not significant (bar 5, Fig. 3K). Results are from *n* = 4 experiments, with measurements taken from at least 175 neurons for each treatment group.

### 3.6. Antibody characterization

In the first instance, antibodies to AT_2_R were evaluated by titration on tissue sections of DRG. Antibody sc-7420 showed immunoreaction with sensory neurons and other structures in which the immunoreactivity diminished with increasing antibody dilution (Supporting Information Fig. S2a–d), and no immunostaining at dilutions greater than 1:500. Abcam antibodies Ab 19134 showed only weak nerve fibres in both pre- and post-fixed tissues, and of the other AT_2_R antibodies (sc-48452, sc-48451 and sc-9040), sc-48452 gave the best immunostaining of DRG neurons and nerve fibres. Pre-absorption of AT_2_R antibody sc-48452, with homologous peptide antigen, abolished all neuronal immunostaining in DRG at 5 × 10^−2^ mg antigen. A gradual return of immunostaining and intensity was achieved with decreasing concentrations of peptide antigen (Supporting Information Fig. S2e–j). Western blotting with extracts of human urinary bladder or normal human nerve using AT_2_R antibody sc-48452 at a dilution of 1:500 revealed a single molecular band of approximately 66 kDa (Supporting Information Fig. S2k).

### 3.7. DRG

**Figure 4:**
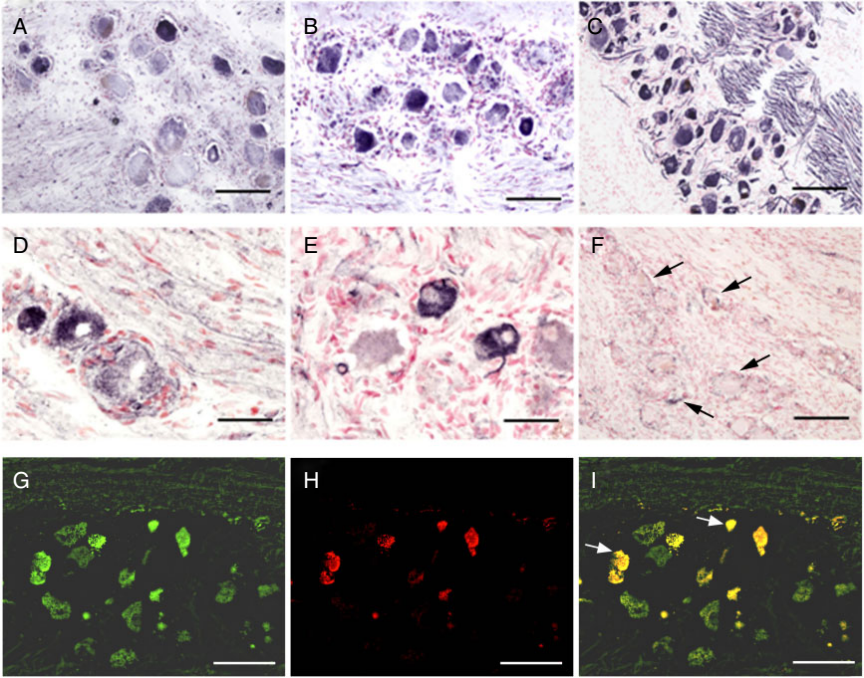
Angiotensin II type 2 receptor (AT_2_R) (A, B) and the neuronal cell marker, neurofilament (C) immunostaining in an avulsed dorsal root ganglion (DRG). AT_2_R-immunoreactive neurons in pre- (D) and post-fixed DRG (E) using antibody sc-48452. AT_1_R immunoreactivity is absent from DRG neuron cell bodies but appears to label vascular structures around the neuronal cells (arrows) (F). Scale bars: A, B = 100 μm; C = 200 μm; D, E = 50 μm, F = 100 μm. Immunofluorescent images of TRPV1 positive neurons in hDRG tissue section (G, green), AT_2_R positive (H, red) and merged image, with arrows indicating co-localization of AT_2_R and TRPV1 (I), bar = 100 μm.

The AT_2_R antibody sc-7420 labelled a sub-population of small-/medium-diameter (≤50 μm) neuronal cells, some of which appeared to be very small and intensely immunoreactive (Fig. 4A and B) in avulsion-injured DRG (*n* = 4, see below). For comparison, a serial section showed a range of sizes of sensory neurons immunostained with nerve marker neurofilaments (Fig. 4C). In pre-fixed DRG, background staining was low and only a few intense positive neurons were present, some with axonal processes, although no AT_2_R-immunoreactive fibres were detected in dorsal nerve roots (Fig. 4D). In post-fixed DRG using antibodies sc-48452 (*n* = 4; age range 21–23 years; trauma-to-surgery delay = 21 days–5 months; 2 women), strong small-/medium-diameter (≤50 μm) neurons were detected along with prominent fine and large calibre nerve fibres within the DRG and dorsal roots (Fig. 4E). Since the AT_2_R antibody sc-48452 showed better immunohistology results for sensory neurons and fibres and appeared specific by pre-absorption and Western blot, it was used for most subsequent studies. In avulsed DRG, the AT_1_ (sc-117) antibody did not label neuronal structures at any of the dilutions tested, but appeared to label vascular structures around the neuronal cells (Fig. 4F).

AT_2_R-immunoreactive, nucleated sensory cells were counted and measured in post-fixed DRG using sc-48452. A total of 519 neurons (*n* = 4, avulsed DRG) were evaluated, and of these, the mean value (mean % total ± SEM) for immunostained small-/medium-diameter (≤50 μm) neurons was 60.4 ± 5.6 and 4.0 ± 1.4 for large (>50 μm) diameter neurons (Fig. 4G). Some very small neurons appeared to be intensely immunoreactive. AT_2_R immunostaining using sc-48452 in post-fixed, control post-mortem DRG (*n* = 4) showed that counts of AT_2_R-immunoreactive, small/medium diameter (≤50 μm) nucleated sensory cells were similar to avulsion injured DRG (post-mortem controls 57.3 ± 5.8). In control hDRG frozen sections, fluorescent immunostaining with the AT_2_R antibody sc48452 showed that AT2R immunostaining neuron counts were similar to the ABC method above; intensely positive AT2R immunoreactivity was co-localized in 41.2 ± 3.7% of TRPV1 positive neurons (Fig. 4G–I: *n* = 4, 172 neurons).

### 3.8. Peripheral nerves

**Figure 5:**
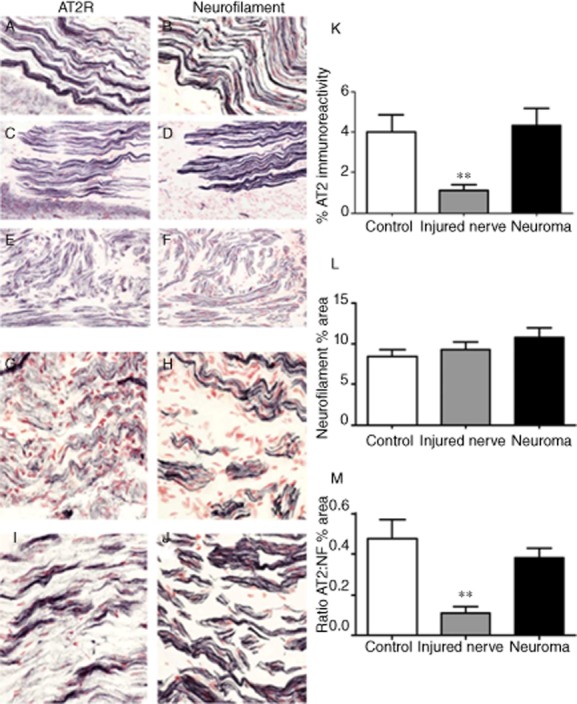
Angiotensin II type 2 receptor (AT_2_R) (A, C, E) and neurofilament (B, D, F) immunostaining of a control peripheral nerve (A, B), proximal injured peripheral nerve (C, D) and neuroma (E, F), magnification ×40. AT_2_R (G, I) and neurofilament (H, J) immunostaining in a neuroma (G, H) and trunk of proximal injured peripheral nerve (I, J), magnification ×40. Image analysis (% area) of AT_2_R (K, **p = 0.003) and neurofilament immunoreactive fibres in control and proximal injured nerve, non-painful and painful neuromas (L). Results (% area) expressed as the ratio AT_2_R : neurofilament (NF), values are mean ± standard error of the mean (M, **p = 0.004).

Post-fixed normal and injured peripheral nerves, and neuromas, all showed AT_2_R-immunopositive fibres (Fig. 5A, C and E). Some painful neuromas and injured nerves showed markedly strong AT_2_R-immunoreactive nerve fibres (Fig. 5G and I). Image analysis of samples of proximal injured nerve (mean % area ± SEM, 1.14 ± 0.25; *n* = 9) showed a significant decrease of AT_2_R immunoreactivity compared with controls (4.00 ± 0.87; *p* = 0.003; *n* = 5; Fig. 5K). This was not the case in neuromas, where levels were comparable to controls (4.35 ± 0.84; *n* = 25; Fig. 5K). There was no significant difference in neurofilament cocktail immunostaining in controls (mean % area ± SEM, 8.46 ± 0.83; Fig. 5L) compared with proximal injured nerves (9.31 ± 0.88; *p* = 0.71) or neuromas (10.84 ± 1.10; *p* = 0.38). Expressing the results as ratio of AT_2_R : neurofilament cocktail in proximal injured nerve (mean % area ± SEM, 0.11 ± 0.03) compared with controls (0.48 ± 0.09; *p* = 0.004; Fig. 5M) also gave a significant decrease. There were no significant differences in the ratios between the control and the neuroma groups (0.38 ± 0.05; *p* = 0.37; Fig. 5M). There were no significant changes of AT_2_R expression in relation to time from injury in DRG or nerve/neuroma specimens.

### 3.9. Skin

In pre-fixed skin, intense AT_1_ immunoreactivity was detected in vascular endothelial/smooth muscle structures, whereas anti-AT_2_R antibodies showed nerve fibres and fascicles in the sub-epidermis and deeper dermis (sc-7420, Supporting Information Fig. S3a; sc-48452, Supporting Information Fig. S3c). These correlated with nerve marker (neurofilaments) in serial sections (Supporting Information Fig. S3b and d). Intra-epidermal nerve fibres were not seen.

### 3.10. Urinary bladder

AT_2_R immunoreactivity was detected in nerve fascicles and scattered single fibres (Supporting Information Fig. S3e and g) correlating with nerve marker (neurofilaments) in the adjacent sections of urinary bladder (Supporting Information Fig. S3f and h).

### 3.11. Intestine

Strongly positive AT_2_R-immunoreactive neurons, some with axonal processes, were detected in the submucous and myenteric plexuses, and a few nerve fibres in the muscularis mucosae of human intestine (Supporting Information Fig. S3i and k). The AT_2_R immunoreactivity appeared to co-localize with nerve marker (neurofilament cocktail) in adjacent sections (Supporting Information Fig. S3j and l). There was no evidence of AT_2_R immunoreactivity in nerve fibres in the mucosa.

## 4. Discussion

This study shows the expression of the AT_2_R in small/medium diameter hDRG and rDRG neurons *in vitro*, and in hDRG tissues, partly co-localized with TRPV1 expression. Application of AngII enhanced capsaicin responses in rDRG neurons, indicating sensitization of the TRPV1 ion channel, and initial studies showed that TRPV1-sensitive cultured hDRG neurons respond to AngII by transient calcium influx. Functional effects of EMA401 pretreatment were observed as dose-dependent inhibition of capsaicin responses in cultured human and rat DRG neurons (IC_50_ = 10 nmol/L), which was reversed in the presence of membrane permeant 8-bromo-cAMP and AngII. Specificity at the AT_2_R was indicated by the reversal of EMA401-mediated inhibition in the presence of AngII and the lack of effect of the AT_1_R antagonist Losartan. Our studies examined the effect of AngII and EMA401 on capsaicin responses in an established *in vitro* hDRG neuron model, requiring the identification of capsaicin sensitive neurons. The dose-related effects of exogenous AngII alone in human and rat DRG neurons, and levels of endogenous AngII *in vitro* and *in vivo* are of importance including any tonic effects, and are part of our ongoing studies.

A major factor determining capsaicin sensitivity is phosphorylation of the capsaicin receptor TRPV1, which is sensitized when phosphorylated and desensitized when dephosphorylated (Bhave et al., 2005). Increased cAMP levels produced by inflammatory mediators such as prostaglandins activate protein kinase A (PKA) in nociceptive afferents, resulting in hyperalgesia, and the direct activation of PKA with cAMP analogues is known to cause behavioural hypersensitivity (Taiwo et al., 1989; Taiwo and Levine, 1991). Accordingly, we observed enhanced capsaicin responses in AngII-treated neurons, which were inhibited in the presence of EMA401. This is likely to involve a GPCR mechanism as AngII application resulted in increased intracellular cAMP, and the inhibitory effect of EMA401 on capsaicin responses was reversed in the presence of forskolin (a source of cAMP) and 8-bromo-cAMP. Together, these findings indicate that EMA401 has an inhibitory effect on capsaicin responses by reducing cAMP. It is thus likely that in situations where AngII is increased, in the periphery/DRG, it may lead to sensitization of TRPV1 and pain. While inhibition of capsaicin responses by 100 nmol/L EMA401 was comparable to maximal inhibitory effects of 1 μmol/L gabapentin and 10 μmol/L morphine, *in vitro* data comparisons should be regarded with caution as they may not reflect *in vivo* mechanisms of action or potency for these compounds. *In vivo* rodent studies utilizing this class of AT_2_R antagonists have shown efficacy in pain models, including neuropathic pain (see Smith, 2011; Smith and Wyse, 2011).

The morphological findings of this study showed that cultured hDRG neurons were positive for Gap43, and double-labelled for the AT_2_R in small diameter neurons, which are known to comprise nociceptors. Following treatment with EMA401, the average neurite length and density were reduced, indicating that neurite branching and length may be affected by the AT_2_R antagonist. AngII treatment significantly enhanced neurite outgrowth, which was completely reversed by EMA401 treatment. This is in agreement with previous studies, which also showed neurite promoting effects of AngII that were blocked with the AT_2_R antagonist PD123319 (Reinecke et al., 2003; Chakrabarty et al., 2008; Alterman, 2010). Neurite vesiculation/disintegration was not observed after EMA401 treatment, indicating the absence of a neurotoxic effect. Treatment with EMA401 appears to have slowed the rate of neurite extension.

Increased cAMP levels observed in AngII-treated neurons were diminished after EMA401 treatment, demonstrating an association with its effect on neurite outgrowth and capsaicin sensitivity. Neurite outgrowth is dependent on cAMP signalling (Kao et al., 2002; Neumann et al., 2002; Murray et al., 2009) and endogenous cAMP levels in neurons decline during development with a concomitant decline in regenerative capacity (Cai et al., 2001). Following nerve injury, NTF expression is up-regulated, resulting in increased cAMP levels, and up-regulation of growth-associated genes actin, tubulin and Gap43 for promotion of axon outgrowth (Gordon, 2009). In our study, cAMP expression was observed to be significantly enhanced in AngII-treated cultures and reduced in the presence of EMA401, indicating a proinflammatory role for AngII in sensory neurons.

The histological results of this study show the presence of AT_2_R protein in neurons of hDRG and nerve fibres of peripheral nerves, skin, urinary bladder and intestine, using immunohistochemistry. The expression of AT_2_R was confirmed with different antibodies and is in accord with previous reports of the presence of AT_2_R mRNA in rDRG and hDRG (Patil et al., 2002), and localization of AT_2_R in small- and medium-sized rDRG neurons (Chakrabarty et al., 2008). Additionally, a significant finding of our study is the co-localization of AT_2_R with a proportion of TRPV1 positive neurons, which corresponds to the AngII-mediated calcium influx in capsaicin sensitive hDRG neurons and indicates a potential role for AT_2_R in nociception. In contrast, AT_1_R immunoreactivity was absent from neurons, but strongly positive in vascular structures, in hDRG and in other tissues. This observation, coupled with the lack of effect of the AT_1_R antagonist Losartan on capsaicin responses in DRG neurons, suggests low level of expression or absence of AT_1_R in hDRG neurons. Western blotting with extracts of human urinary bladder or nerve using AT_2_R antibody sc-48452 revealed a single molecular band of 66 kDa similar in magnitude to that previously reported for extracts of human myometrium, where the molecular mass of AT_2_R was 68 ± 4.6 kDa (Servant et al., 1994). Antibody sc-48452 showed best immunohistology results and appeared specific by pre-absorption and Western blot.

In post-mortem control human DRG, AT_2_R expression was intense in a large proportion of small/medium diameter neurons and was similar to avulsion-injured DRG neurons (the latter is the equivalent of ‘central axotomy’ or spinal nerve root lesion proximal to the DRG). In contrast, AT_2_R levels were reduced in human nerve segments proximal to injury (lesion distal to the DRG or ‘peripheral axotomy’), but they were preserved or high in painful neuromas, suggesting maintained expression in regenerating nerve fibres. The regulation of AT_2_R expression in injured human sensory neurons, and the role of target-organ derived factors after nerve injury, deserves further study. Normal skin showed AT_2_R positive nerve fascicles in deep dermis, sub-epidermal fibres and nerve fibres associated with sweat glands and neurovascular structures. In the urinary bladder, AT_2_R positive nerve fibres were observed in nerve fascicles and as scattered sub-urothelial fibres. In bowel, AT_2_R immunostaining was present in nerve fibres of the mucosa and circular and longitudinal muscle layers, and also in a subset of myenteric plexus neurons. The localization of AT_2_R in human tissues suggests a potential role in diverse clinical pain states, including somatic and visceral disorders.

The AT_2_R was shown to be up-regulated in the skin of neonatal rats following injury (Viswanathan and Saavedra, 1992) and in PC12 cells by the AT_2_R agonist CGP42112 (Abadir et al., 2011). Oestrogen was shown to affect axonal sprouting in cultured DRG neurons by locally produced AngII via up-regulation of AT_2_R, and proposed to underlie the increased prevalence of pain conditions common in pre-menopausal women, such as migraine, painful bladder syndrome/interstitial cystitis, and irritable bowel syndrome (Chakrabarty et al., 2008). As NGF-induced neurite outgrowth in rDRG cultures was not affected by AT_2_R blockade, EMA401, while sharing features in common with anti-NGF antibodies, may provide improved safety compared with anti-NGF therapy. AT_2_R antagonists could be particularly useful in the treatment of chronic pain and hypersensitivity associated with abnormal nerve sprouting.

AngII may act as a sensory neurotransmitter and on up-regulated AT_2_R in sensory neurons by autocrine, paracrine and systemic mechanisms, particularly in relation to vascular innervation, via peripheral and central nerve terminals (including pre-synaptic nerve terminals), and DRG neuronal cell bodies. In the CNS, AngII and its metabolite AngIII both act at the AT_1_R and the AT_2_R (Martens et al., 1996; Zini et al., 1996; Wright et al., 2003; Pelegrini-da-Silva et al., 2005), playing a role in pain mechanisms (see Georgieva and Georgiev, 1999, Pelegrini-Da-Silva et al., 2005, Sakagawa et al., 2000; Marques-Lopes et al., 2009, 2010), by the distinct expression of AT_1_R in neurons involved in descending pain modulation (Marques-Lopes et al., 2009), and the conversion of AngII to AngIII (Pelegrini-da-Silva et al., 2009). However, our study relates to the effects of EMA401 on peripheral mechanisms, since it does not show significant CNS distribution. Oral gavage dosing of ^14^C-EMA401 at 1 mg/kg in rats did not show detectable radioactivity levels in the brain at the lower limit of quantitation (3.66 ng EMA401/g tissue), and there were no significant CNS-related side effects in either rats dosed at 1000 mg/kg oral gavage, or in humans during the phase 1 programme (Spinifex, unpublished data).

In conclusion, AT_2_R antagonist EMA401 treatment resulted in a dose-related functional inhibition of capsaicin responses, and reduced neurite length, in cultured human and rat DRG neurons. The distribution of AT_2_R in human DRG nociceptors, co-localized with TRPV1, along with the functional *in vitro* studies, indicates that novel drugs such as EMA401 may inhibit pain perception in clinical disorders.

## Summary stating conclusions of study

The angiotensin II receptor subtype 2 (AT_2_R) is expressed in nociceptive nerve fibres in rat and human tissues; AT_2_R antagonist EMA401 inhibits capsaicin responses and neurite outgrowth in human and rat cultured DRG neurons.

## Supporting Information

**Figure S1** Structure of EMA401 EMA401 is the (S)-enantiomer of EMA400, prepared as the racemate, and also referred to as PD-126,055, is a member of the 1,2,3,4-tetrahydroisoquinoline-3-carboxylic acid class of AT_2_ receptor antagonists.

**Figure S2** Top left panel: DRG immunostained with a range of dilutions of AT_2_R antibody sc-7420 at 1:25 (a), 1:50 (b), 1:100 (c), and 1:200 (d) dilutions, scale bars a–d = 500 μm. Top right panel. Serial sections of DRG nerve roots immunostained with AT_2_R antibodies sc-48452 with no peptide (e) or with peptide at 5 × 10^−2^ (f), 5 × 10^−3^ (g), 5 × 10^−4^ (h), 5 × 10^−5^ (i) or 5 × 10^−6^ (j) mg/mL antibody at a dilution of 1:100; Scale bars e–j = 100 μm. Bottom left panel: Western blots of PBS bladder (lane 1) and normal nerve (lane 2) extract using the AT_2_R sc- 48452 antibody at 1:500 (k); The positions of the 76 and 52 kDa molecular weight markers are indicated.

**Figure S3** AT_2_R immunostaining in human skin (a, magnification ×80; c, magnification × 40), human urinary bladder (e, g, magnification × 40) and human intestine (i, k, magnification × 40). Serial sections (b, magnification × 80; d, f, h, j, l, magnification × 40) are immunostained with nerve marker (neurofilaments).

**Method S1** Supplementary details for calcium imaging.

**Method S2** Detailed protocol for Western blotting.
