# Supplementary material for: Angiotensin II type 2 receptor (AT2R) localization and antagonist-mediated inhibition of capsaicin responses and neurite outgrowth in human and rat sensory neurons
Source: Eur J Pain. 2012 Dec 17;17(7):1012–26. doi: 10.1002/j.1532-2149.2012.00269.x (PMC3748799; doi:10.1002/j.1532-2149.2012.00269.x)
Supplement: Supplementary file 1 [file ejp0017-1012-SD1.zip › ejp_269_sm_figureS1.docx]

**Figure 1S**


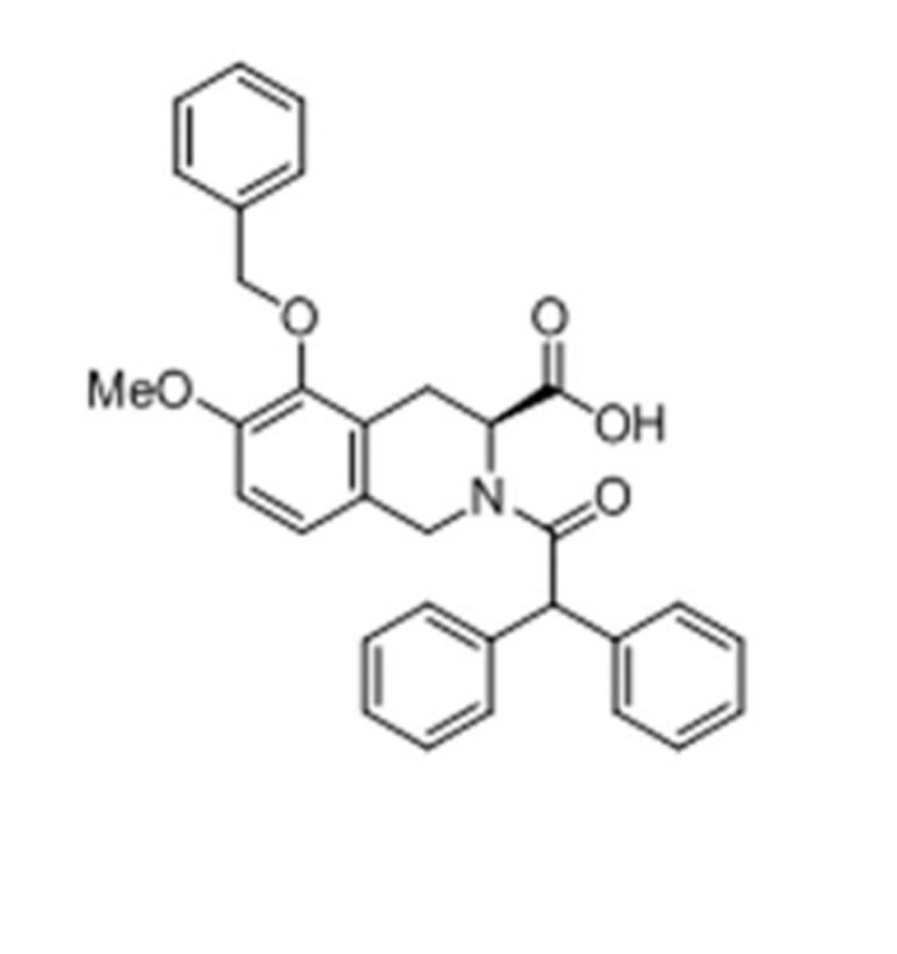


**Figure 1S.** Structure of EMA401 EMA401 is the (S)-enantiomer of EMA400, prepared as the racemate, and also referred to as PD-126,055, is a member of the 1,2,3,4-tetrahydroisoquinoline-3-carboxylic acid class of AT_2_ receptor antagonists.
