# Supplementary material for: Angiotensin II type 2 receptor (AT2R) localization and antagonist-mediated inhibition of capsaicin responses and neurite outgrowth in human and rat sensory neurons
Source: Eur J Pain. 2012 Dec 17;17(7):1012–26. doi: 10.1002/j.1532-2149.2012.00269.x (PMC3748799; doi:10.1002/j.1532-2149.2012.00269.x)
Supplement: Supplementary file 1 [file ejp0017-1012-SD1.zip › ejp_269_sm_figureS2.docx]

**Figure 2S**


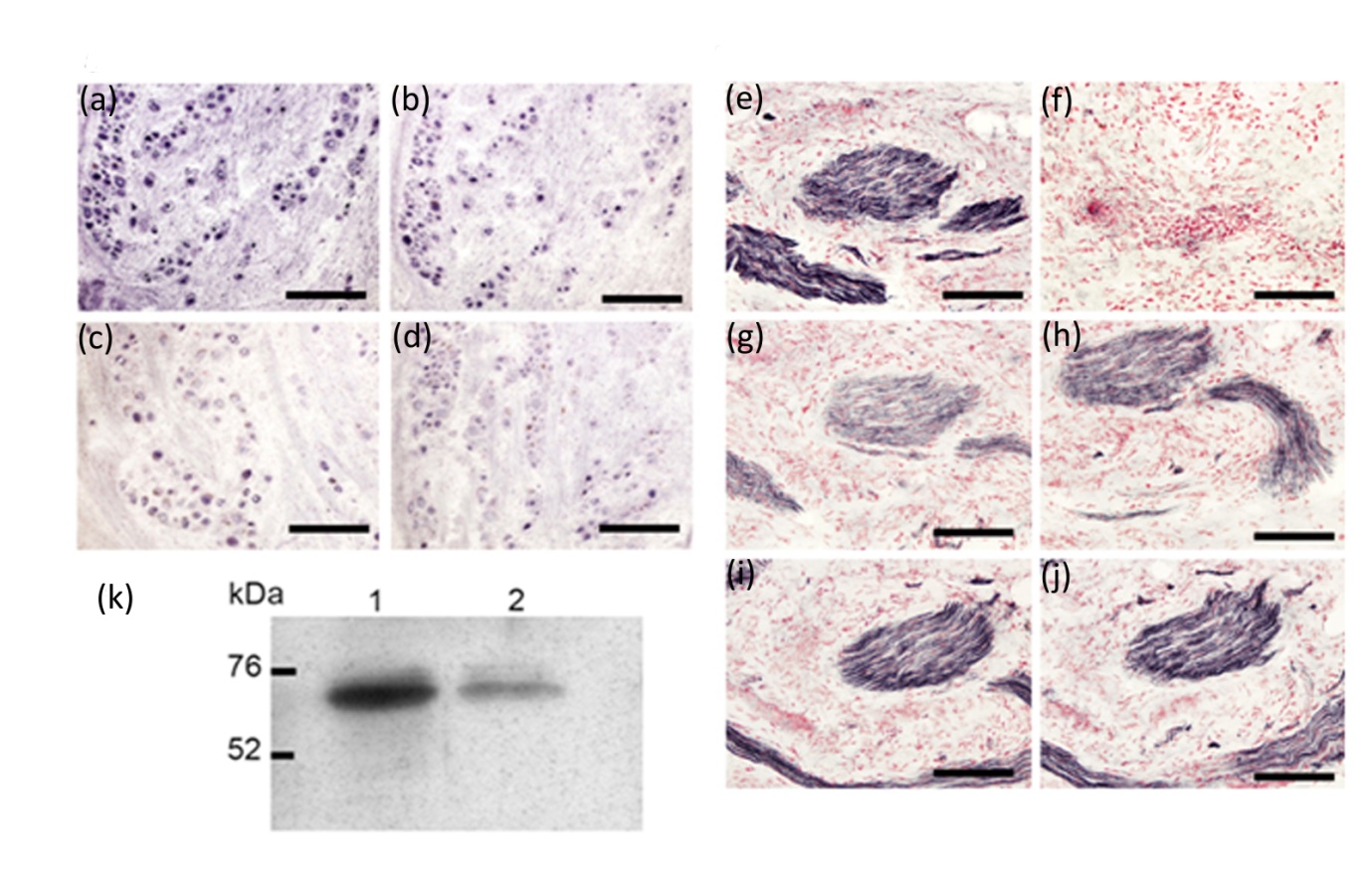


**Figure 2S.**  Top left panel. DRG immunostained with a range of dilutions of AT_2_R antibody sc-7420 at 1:25 (a), 1:50 (b), 1:100 (c), and 1:200 (d) dilutions, scale bars a-d = 500 µm. Top right panel. Serial sections of DRG nerve roots immunostained with AT_2_R antibodies sc-48452 with no peptide (e) or with peptide at 5 x 10^-2^ (f), 5 x 10^-3^ (g), 5 x 10^-4^ (h), 5 x 10^-5^ (i) or 5 x 10^-6^ (j) mg/ml antibody at a dilution of 1:100; Scale bars e-j =100 µm. Bottom left panel. Western blots of PBS bladder (lane 1) and normal nerve (lane 2) extract using the AT_2_R sc- 48452 antibody at 1:500 (k); The positions of the 76 and 52 kDa molecular weight markers are indicated.
