# Supplementary material for: Angiotensin II type 2 receptor (AT2R) localization and antagonist-mediated inhibition of capsaicin responses and neurite outgrowth in human and rat sensory neurons
Source: Eur J Pain. 2012 Dec 17;17(7):1012–26. doi: 10.1002/j.1532-2149.2012.00269.x (PMC3748799; doi:10.1002/j.1532-2149.2012.00269.x)
Supplement: Supplementary file 1 [file ejp0017-1012-SD1.zip › ejp_269_sm_figureS3.docx]

**Figure 3S**


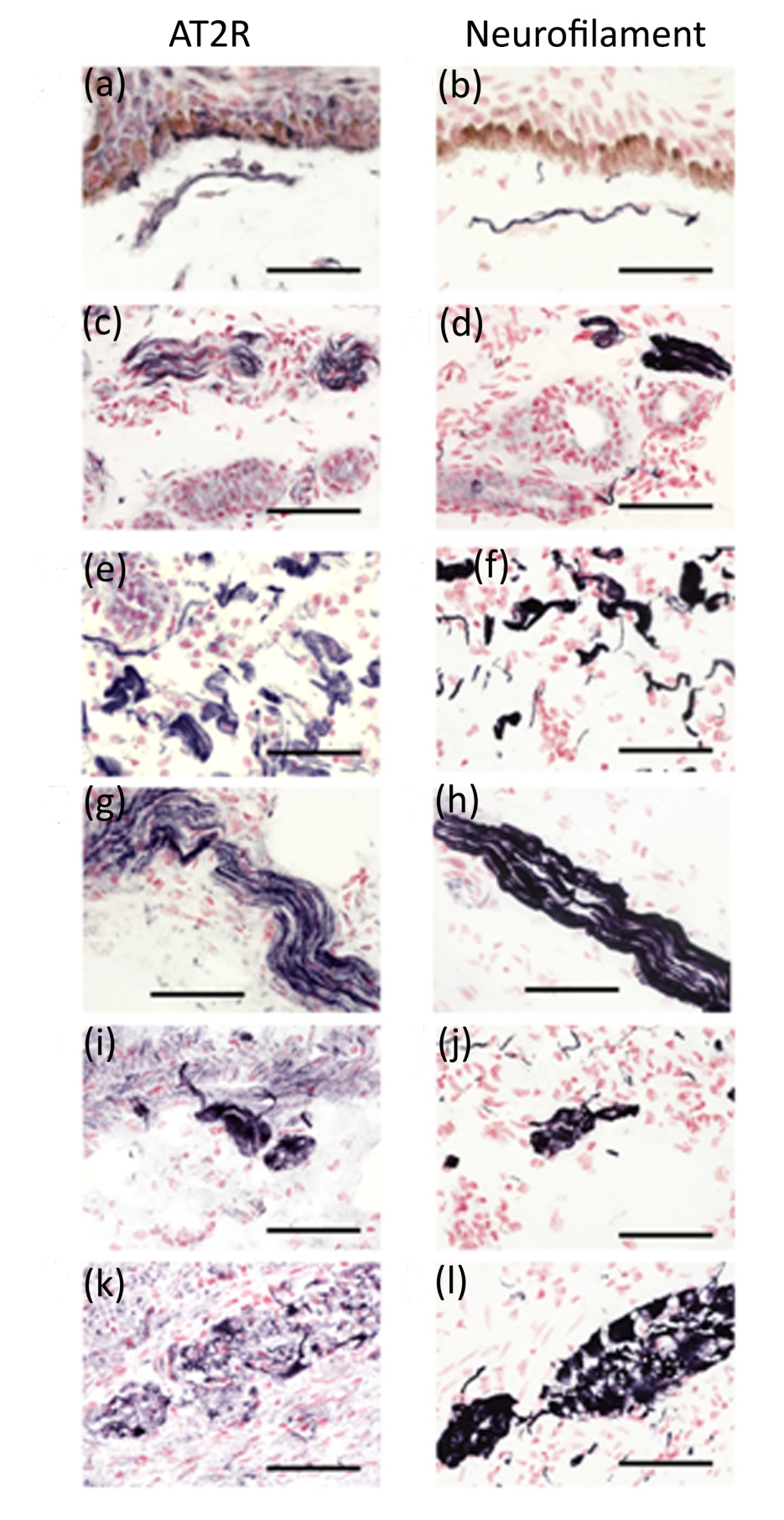


**Figure 3S.** AT_2_R immunostaining in human skin (a, magnification x80; c, magnification x40), human urinary bladder (e, g, magnification x40) and human intestine (i, k, magnification x40). Serial sections (b magnification x80, d, f, h, j, l, magnification x40) are immunostained with nerve marker (neurofilaments).
