# Supplementary material for: Angiotensin II type 2 receptor (AT2R) localization and antagonist-mediated inhibition of capsaicin responses and neurite outgrowth in human and rat sensory neurons
Source: Eur J Pain. 2012 Dec 17;17(7):1012–26. doi: 10.1002/j.1532-2149.2012.00269.x (PMC3748799; doi:10.1002/j.1532-2149.2012.00269.x)
Supplement: Supplementary file 1 [file ejp0017-1012-SD1.zip › ejp_269_sm_Methods S1.docx]

**Methods S1** (Supplementary details for calcium imaging).

Images were captured every 2 s in each of the three channels-phase, 340 and 380 nm, and recordings of intracellular changes in bound and unbound Ca^2+^ ratio were obtained before, during and after the addition of test compounds. This provided baseline recordings as well as intracellular changes in Ca^2+^ levels in response to added compounds. Cells were uniformly loaded with the dye and no intracellular compartmentalisation of the loaded dye was observed. Images were acquired with a Hamamatsu Orca CCD Camera and analysed with AQM Advance Kinetic imaging software. Individual cells under study were highlighted as regions of interest for calculating the mean ratios of bound to unbound calcium within the area of interest. In each experiment, neurons were exposed to capsaicin for a maximum of two applications only, first to identify capsaicin sensitivity and second to test the effect of the added drugs after the washout period. As capsaicin stimulation is known to cause desensitisation, we employed a protocol whereby a minimum concentration (200 nM) and brief period of application (15 s) was used to identify a capsaicin-sensitive neuron (demonstrating a rapid increase in 340/380 ratio and sustained response), which was followed by washout of medium and a rest period of 30 minutes.
