# Supplementary material for: Angiotensin II type 2 receptor (AT2R) localization and antagonist-mediated inhibition of capsaicin responses and neurite outgrowth in human and rat sensory neurons
Source: Eur J Pain. 2012 Dec 17;17(7):1012–26. doi: 10.1002/j.1532-2149.2012.00269.x (PMC3748799; doi:10.1002/j.1532-2149.2012.00269.x)
Supplement: Supplementary file 1 [file ejp0017-1012-SD1.zip › ejp_269_sm_Methods S2.docx]

**Methods S2 (Detailed protocol for Western Blotting)**

Homogenates (30 µg total protein) and molecular weight markers (RPN 800 GE Healthcare) were combined with SDS sample buffer and incubated at 100^o^C for 5 minutes before loading on 10% SDS-polyacrylamide gels. After electrophoresis, proteins were transferred to PVDF membranes (Amersham Life Science Ltd, Buckinghamshire, U.K.), using a semi-dry transblotter at a constant 170 mA for 16 hours at 4°C. Non-specific binding sites were blocked by incubating in 4% (w/v) casein in a solution of PBS containing 0.1% (v/v) Tween 20 for 1 hour. The primary antibodies were prepared in 1.5 % casein PBS/Tween buffer and incubated with membranes for 2 h at room temperature. After washing in wash buffer, sites of attachment of primary antibodies were detected using immunoperoxidase reagents (Vector Laboratories, Peterborough UK). Immunoreactivity was visualised on Hyperfilm film after treatment with ECL-plus Western blotting detection system (Amersham Life Science Ltd, Buckinghamshire, UK).
